# A comparative meta-analysis of the efficacy and safety of arthroscopic versus open surgery in patients with lateral epicondylitis

**DOI:** 10.1016/j.jor.2024.07.018

**Published:** 2024-07-25

**Authors:** Maher Ghandour, Diaa AL Salloum, Mohamad Houssein Jaber, Ghadi Abou Orm, Ali Ghosn, Sadek Jaber, Hicham Abd El Nour, Anthony Chalfoun, Tanios Dagher, Bashour Hanna

**Affiliations:** a Department of Orthopedic Surgery, CHU Grenoble Alpes, Grenoble, France; b Department of Orthopedic Surgery, APHP Henri Mondor, Paris, France; c Department of Orthopedic Surgery, Lebanese American University Medical Center-Rizk Hospital, Beirut, Lebanon; d Department of Orthopedic Surgery, Saint Georges University Medical Center, Beirut, Lebanon; e Department of Orthopedic Surgery, Hopital Ambroise-Paré, Paris, France; f Orthopaedic Department, CHU Grenoble Alpes, Grenoble, France

**Keywords:** Tennis elbow, Lateral epicondylitis, Arthroscopic, Open

## Abstract

**Background:**

Lateral epicondylitis frequently necessitates surgical management when non-surgical treatments are ineffective. Anecdotal evidence suggests comparable efficacy between arthroscopic and open surgical repair; however, it is limited by the scarcity of data. This meta-analysis compares between both procedures regarding functional recovery, pain intensity, complications, and return-to-work time.

**Methods:**

A detailed systematic review and meta-analysis of research published until February 2024 were performed, comparing arthroscopic and open surgery methods for lateral epicondylitis. The studies were sourced from PubMed, Scopus, Web of Science, Cochrane Library, and Google Scholar. The included studies examined outcomes such as functional recovery, pain intensity, complication rates, and time to return to work. The risk of bias was evaluated using the Cochrane tool for randomized studies and the ROBINS-I tool for non-randomized studies.

**Results:**

The meta-analysis included 19 studies with a total of 20,409 participants. The analysis found no significant differences in postoperative Disabilities of the Arm, Shoulder, and Hand (DASH) scores (Mean Difference [MD] = 0.06; 95 % Confidence Interval [CI]: 0.81 to 0.94; P = 0.89) or Mayo Elbow Performance Scores (MD = 0.31; 95 % CI: 2.33 to 2.95; P = 0.80) between the arthroscopic and open surgical methods. The rates of good-to-excellent recovery, surgical failures, and complications were similar across both techniques. Nevertheless, arthroscopic surgery was associated with a significantly shorter return-to-work period (MD = −1.64 months; 95 % CI: 2.60 to −0.68; P = 0.001) and a temporary increase in grip strength six months after surgery (MD = −1.50 kg; 95 % CI: 2.67 to −0.33; P = 0.012).

**Conclusions:**

Arthroscopic and open release techniques for lateral epicondylitis provide similar functional outcomes and complication rates. However, arthroscopic surgery may allow for a quicker return to work, suggesting a potential advantage in the early postoperative period. These findings highlight the need for individualized surgical decision-making based on patient-specific factors and surgeon expertise.

## Highlights

• This meta-analysis of 20,488 patients with lateral epicondylitis compared arthroscopic to open surgery.
• Both arthroscopic and open surgery techniques showed similar functional recovery and complication rates.
• Arthroscopic surgery correlates with shorter return-to-work times and improved grip strength at six months post-operation.

## 1 Introduction

Lateral epicondylitis, often known as tennis elbow,^1^ is a common musculoskeletal condition marked by pain and sensitivity in the lateral elbow area, mainly at the origin of the extensor carpi radialis brevis tendon.^2^ It predominantly affects individuals involved in activities that require repetitive wrist extension and forearm supination. This includes not only athletes but also professionals like carpenters and plumbers.

The pathogenesis of lateral epicondylitis involves microtears and degeneration of the extensor carpi radialis brevis tendon, leading to a failed healing response.^3^ Histopathological studies have shown that the condition is characterized by angiofibroblastic hyperplasia, indicating a chronic degenerative process rather than an acute inflammatory condition.^4^ The underlying mechanisms are thought to include repetitive mechanical overload, microtrauma, and subsequent tendinosis.^5^

Despite its frequent occurrence, the best approach to manage lateral epicondylitis is still widely debated, especially regarding the decision to use arthroscopic versus open surgical methods.^6^, ^7^, ^8^

Non-operative treatments, including physiotherapy, corticosteroid injections, and activity modification, are initially recommended for most patients.^9^ However, when these conservative measures fail, surgical intervention becomes a consideration. The surgical approaches for lateral epicondylitis have evolved significantly over the years, from open debridement and release to less invasive methods such as arthroscopic surgery.^10^ Each technique has its proponents, underpinned by various claims regarding efficacy, recovery time, and complication rates.

The literature presents a mixed picture with some studies suggesting that arthroscopic release offers quicker recovery and reduced pain post-surgery,^11^^,^^12^ while others find no substantial differences between the two techniques in terms of functional outcomes and long-term relief.^13^^,^^14^ These discrepancies pose challenges for clinicians in deciding the best surgical approach, highlighting the need for a comprehensive review and meta-analysis to synthesize existing evidence.^6^^,^^15^ A recent systematic review compared both approaches for the treatment of tennis elbow. Although the study included 37 studies, denoting comparable success rate between both procedures, their conclusions were based on the quantitative analysis of only three to four studies with substantial heterogeneity.^6^

Given the ongoing uncertainty and the potential impact of surgical choice on patient outcomes, this systematic review and meta-analysis aim to compare the effectiveness and safety of arthroscopic versus open release techniques in the treatment of lateral epicondylitis. By integrating findings from diverse studies, this research endeavors to provide clearer guidance for clinicians on the optimal surgical management of tennis elbow.

## 2 Materials and methods

### 2.1 Study design

This systematic review and meta-analysis adhered to the 2020 updated Preferred Reporting Items for Systematic Reviews and Meta-Analyses (PRISMA) guidelines.^16^

### 2.2 Literature search

A thorough search was performed across several electronic databases including PubMed, Scopus, Web of Science, the Cochrane Library of Randomized Trials (CENTRAL), and Google Scholar, spanning their inception to February 22nd, 2024. In line with recent guidelines,^17^ we limited our Google Scholar results to the first 200 entries for screening. Our search strategy targeted studies that contrast arthroscopic with open surgery techniques in treating lateral epicondylitis, using terms and Medical Subject Headings (MeSH) such as “lateral epicondylitis,” “tennis elbow,” “arthroscopy,” “open surgery,” and “comparative studies.” For a comprehensive search strategy, refer to Table S1. No language limitations were imposed. Further accuracy in our search was ensured by manually reviewing references in selected studies, checking PubMed's related articles, and performing a manual Google search using the study keywords.^18^ Exclusions were made for non-original studies (e.g., reviews, commentaries), studies on conditions other than lateral epicondylitis, studies lacking a comparison group, duplicates, and those with overlapping patient data.

### 2.3 Selection strategy

We used a detailed PICOS (Population, Intervention, Comparison, Outcomes, and Study Design) framework to define eligibility criteria.^19^ We included observational and experimental comparative studies, both randomized and non-randomized, involving patients with lateral epicondylitis requiring surgical intervention, comparing arthroscopic to open surgical methods, and documenting any post-surgical clinical outcomes. We verified the absence of overlap by reviewing the period of patient recruitment, country, and demographic details such as age and gender.

### 2.4 Data collection and outcome measures

The data collection form, created in Microsoft Excel by the lead author, was divided into three sections: the first recorded details about the studies and participants, the second focused on outcomes like functional elbow recovery measured via DASH, QuickDASH, and Mayo Elbow Performance Score (MEPS), and the third section evaluated the risk of bias in the studies.

The primary outcome focused on various aspects of elbow function recovery. This encompassed ratings from good to excellent, as well as instances of poor recovery, failure, and instances necessitating further surgeries or revisions. The evaluation of functional recovery utilized the DASH, QuickDASH, and MEPS scales. A good recovery was classified by scores of 20–39 on the DASH, 6–15 on the QuickDASH, and 75–89 on the MEPS.^20^^,^^21^ An excellent recovery was indicated by a DASH score below 20, a QuickDASH score between 0 and 5, and a MEPS score ranging from 90 to 100 ^20, 21^. Secondary outcomes were comprised of recurring pain, defined as pain resurfacing after at least one month of being pain-free, elbow motion range during flexion and extension, grip strength, postoperative pain measured via the visual analogue scale (VAS), arthroscopic observations of intra-articular loose bodies and noticeable synovitis, times related to returning to work and staying off work, duration of the operation, and the rate of complications. The final section addressed the risk of bias evaluation for the included studies.

### 2.5 Risk of bias assessment

Bias in randomized controlled trials was evaluated using the Cochrane Collaboration's Risk of Bias tool (RoB-2), while non-randomized studies were assessed through the ROBINS-I tool. Discrepancies were resolved by consensus or third-party adjudication.

### 2.6 Data synthesis and analysis

All statistical evaluations were conducted using STATA Software (Version 18, Stata Corp, USA). Prior to the meta-analysis, necessary data transformations were applied to standardize the reporting by converting median/range or interquartile range data into mean and standard deviation, employing validated equations.^22^^,^^23^

For aggregating outcomes, the last observation carried forward (LOCF) method was used to address data heterogeneity across different follow-up periods and to reduce the risk of data omission.^24^ Both the log odds ratio (logOR) for binary outcomes and the mean difference (MD) for continuous outcomes were calculated, and analyzed using a random-effects model applying the restricted maximum likelihood (REML) method. We examined Galbraith plots to detect any outliers, discarding data confirmed as inaccurate.

The random-effects model was chosen due to expected clinical variability across the studies. We assessed heterogeneity using the I^2^ statistic, categorizing 25 %, 50 %, and 75 % as low, moderate, and high heterogeneity, respectively.^25^ Further analysis included subgroup examinations based on follow-up duration to probe potential heterogeneity sources and to observe variations in treatment effects over time.

Robustness of the results was tested through sensitivity analyses that involved sequentially excluding studies to observe the effects on overall results, particularly under conditions of notable heterogeneity. Due to the limited number of studies in each outcome analysis (<10), conducting publication bias assessment using funnel plots and Egger's test for asymmetry was impractical.

## 3 Results

### 3.1 Literature search and screening results

The search yielded 424 citations, with 165 identified as duplicates. Of the remaining 259, 235 were excluded at the title/abstract screening stage (Fig. 1). Full-text screening was conducted for the remainder, with one article inaccessible and five excluded for lacking original data. A manual search added one more study, bringing the total to 19 studies included in the analysis.^11^, ^12^, ^13^, ^14^^,^^26^, ^27^, ^28^, ^29^, ^30^, ^31^, ^32^, ^33^, ^34^, ^35^, ^36^, ^37^, ^38^, ^39^, ^40^

**Fig. 1:**
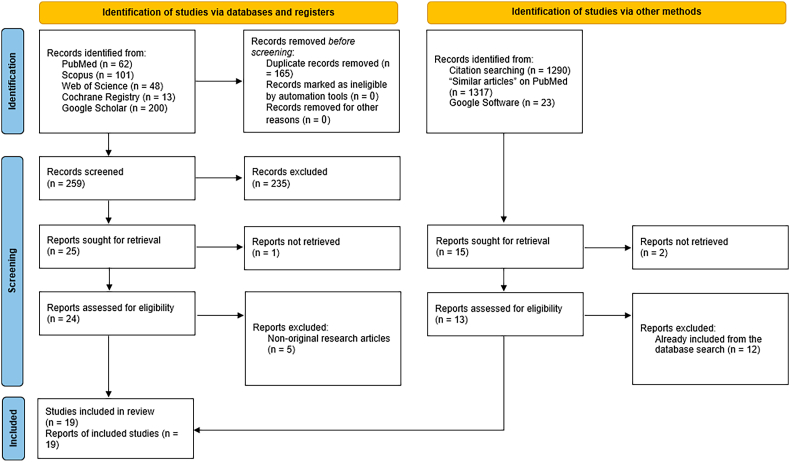
PRISMA diagram showing the results of the literature search and screening processes.

### 3.2 Baseline characteristics of included studies

Table 1 offers an exhaustive breakdown of the attributes of the studies included. The majority of these studies were conducted in the Republic of Korea and the United States, each hosting four studies, followed by two studies each in Canada, Norway, and Spain. Most of the studies (11) were retrospective cohorts, with the remainder comprising six randomized controlled trials, one prospective cohort, and one case-control study. Detailed information on the study populations, including age and gender distribution, is also provided in Table 1. A total of 20,409 patients diagnosed with lateral epicondylitis were part of these studies, with 2770 receiving arthroscopic release treatment and 17,639 undergoing open surgery. The duration of follow-up varied widely, ranging from 2 weeks to nearly 94 months.

**Table 1:** Baseline characteristics of included studies comparing arthroscopic to open release in patients with lateral epicondylitis.

Author (YOP)	Country	Design	YOI	Population	Sample^a^		Description		M/F		Age		FU (mo)	
					AG	OG	AG	OPG	AG	OG	AG	OG	AG	OG
**Bhandari et al (2020)**^26^	USA	RC	2010–2017	Symptomatic patients after 9 months of conservative treatments (nonsteroidal medications, steroid injections, and physical therapy)	30	42	Arthroscopic debridement	Open tenotomy and reinsertion	32/40		28–67		1.5	1.5
**Clark et al (2018)**^27^	Canada	RCT	2002–2014	Patients >16 years with a minimum of 6 months of nonoperative management for lateral epicondylitis	38	37	Arthroscopic lateral release	Open release	19/18	22/16	45.6 (1.1)	46.9 (1.2)	0.46	0.46
**Kim et al (2018)**^28^	Republic of Korea	RC	2011–2015	Patients with lateral epicondylitis refractory to 6 months of conservative treatment (NSAIDs, corticosteroid injections, exercise, and elbow brace)	34	34	Arthroscopic surgery	Open surgery (Nirschl)	16/18	22/12	48 (8.1)	49 (7.8)	24	24
**Kim et al (2005)**^29^	Republic of Korea	RC	–	Patients with lateral epicondylitis unresponsive to conservative treatment (NSAIDs, steroid injections, physical exercise)	17	14	Arthroscopic release	Open surgery	–	–	46	42	14	14
**Kim et al (2008)**^30^	Korea	RC	–	Patients with recalcitrant lateral epicondylitis	13	21	Arthroscopic release	Open release	–	–	–	–	12	12
**Kundu et al (2022)**^13^	India	RCT	2019–2020	Patients with recurrent episodes of recalcitrant lateral epicondylitis	15	16	Arthroscopic debridement of ECRL, ECRB, and ECRC	Open debridement of ECRL, ECRB, and ECRC	7/9	9/6	41.53 (7.07)	41.26 (5.93)	10.86 (3.13)	12.2 (2.98)
**Kwon et al (2017)**^14^	Republic of Korea	RC	2008–2014	Patients who did not improve after conservative treatment (steroid injections)	30	29	Arthroscopic debridement	Open (Nirschl) procedure	21/8	19/7	49.3 (36.74)	51.8 (41.75)	31 (12.5)	28.5 (14.6)
**Leiter et al (2016)**^32^	Canada	RCT	–	Patients who did not improve after conservative treatment (not clearly described) for at least 6 months	34	34	Arthroscopic lateral release	Open lateral release	21/13	19/15	45 (6.9)	47.1 (6.7)	12	12
**López-Alameda et al (2022)**^33^	Spain	RC	2014–2017	Patients with resistant lateral epicondylitis	27	20	Arthroscopic release	Open surgery (Wolff technique)	22/25		47.44 (8.07)	46.05 (8.08)	12	12
**Meknas et al (2013)**^34^	Norway	RCT	2006–2007	Patients with tendinosis in the lateral epicondyle of the elbow	13	11	Arthroscopic microtenotomy	Open release	13/11		46.2 (13.1)	49.2 (11.6)	68.4 (6.2)	75.5 (8.1)
**Moran et al (2023)**^35^	USA	RC	2010–2019	Patients diagnosed with lateral epicondylitis in the MUExtr database	2141	17139	Arthroscopic debridement	Open debridement	968/1173	7116/10023	47.68 (8.75)	48.67 (8.49)	60	60
**Peart et al (2004)**^36^	USA	RC	1997–2002	Patients with resistant lateral epicondylitis	33	54	Arthroscopic release	Open procedure	–	–	–	–	22	16
**Solheim et al (2013)**^11^	Norway	CC	2002–2005	Patients operated upon for lateral epicondylitis	225	80	Arthroscopic release of ECRB	Open tendon release	105/120	44/36	46 (8)	46 (8)	50 (10)	49 (9)
**Yan et al (2009)**^12^	China	RCT	2006–2008	Patients with recalcitrant lateral epicondylitis	15	13	Arthroscopic Nirschl procedure	Open (Nirschl) procedure	–	–	–	–	17.4 (4–32)	
**Zayed et al (2020)**^40^	Egypt	PC	2015–2018	Patients with resistant tennis elbow	15	15	Arthroscopic release	Open release	9/6	8/7	37.47	34.6	13.46 (2.19)	13.8 (2.21)
**Rubenthaler et al (2005)**^37^	Germany	RC	1992–1995	Patients with chronic lateral epicondylitis	20	10	Arthroscopic release	Open Hohmann procedure	11/9	7/3	46.8	54.2	93.6	91.5
**Szabo et al (2006)**^39^	USA	RC	1997–2002	Patients undergoing operative treatment for recalcitrant lateral epicondylitis	41	38	Arthroscopic release of ECRB	Open release of ECRB	29/12	21/16	45.5	46.1	47.3	53.5
**Lee et al (2018)**^31^	Republic of Korea	RCT	2010–2015	Patients with recalcitrant lateral epicondylitis	24	22	Arthroscopic release of ECRB	Open release (radiofrequency-based microtenotomy)	11/13	8/14	51.25 (8.57)	51.59 (5.75)	24	24
**Stapleton and Baker (1996)**^38^	Spain	RC	1989–1994	Patients with lateral epicondylitis unresponsive to conservative treatment (not clearly reported)	5	10	Arthroscopic release	Open release	–	–	–	–	3	3

a The provided sample size is that of examined elbows and not of recruited patients to account for laterality. YOP: year of publication; RC: retrospective cohort; PC: prospective cohort; RCT: randomized controlled trial; CC: case-control; AG: arthroscopic group; OG: open group; ERCB: extensor carpi radialis brevis; M/F: number of males/females; FU: follow-up; mo: month; UK: United Kingdom; USA: United States of America; NSAIDs: nonsteroidal anti-inflammatory drugs.

### 3.3 Risk of bias

The risk of bias evaluation is summarized in Fig. 2, Fig. 3. Six randomized controlled trials (RCTs) were assessed using Cochrane's RoB-2 tool. This assessment revealed that three RCTs exhibited a high risk of bias, two RCTs presented some concerns, and only one RCT was found to have a low risk of bias. For non-randomized studies, three were determined to have a low risk of bias, whereas the other ten exhibited a moderate risk, largely due to confounding factors and reporting biases.

**Fig. 2:**
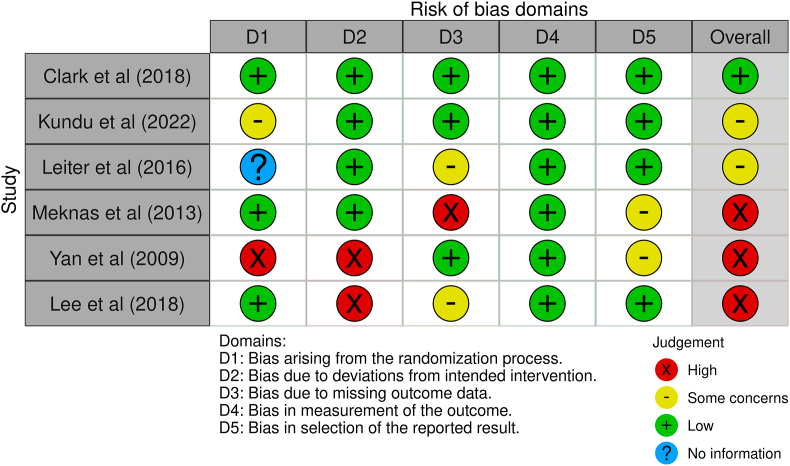
The risk of bias assessment of randomized controlled trials using Cochrane's RoB-2 tool.

**Fig. 3:**
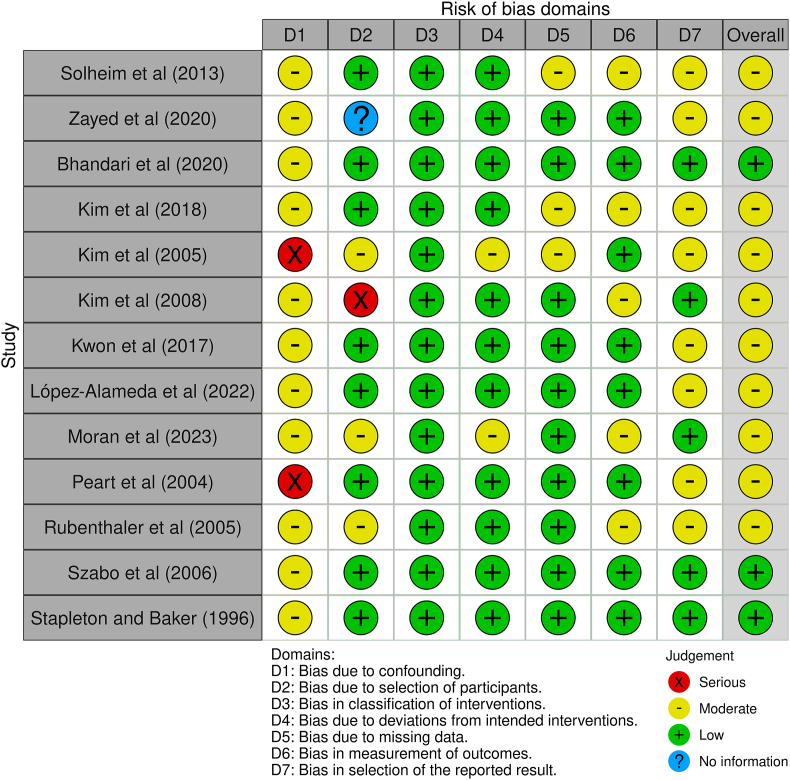
The risk of bias assessment of non-randomized trials using ROBINS-I tool.

### 3.4 Functional recovery

#### 3.4.1 DASH score

No significant change the postoperative DASH score was observed between the arthroscopic and open approaches [7 studies, MD = 0.06; 95%CI: 0.81: 0.94] (Fig. 4). Although moderate heterogeneity was observed, it was statistically insignificant [I^2^ = 51.52 %, *P* = 0.05].

**Fig. 4:**
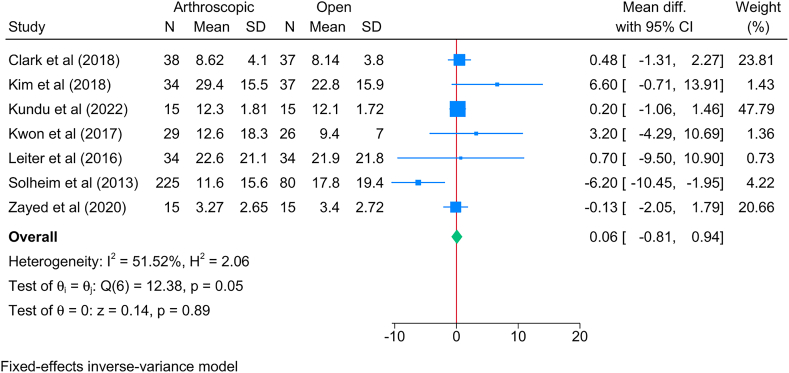
Forest plot showing the difference in postoperative DASH score between arthroscopic and open lateral release in patients with lateral epicondylitis.

The subgroup analysis based on time showed a significant reduction in the DASH score in favor of the arthroscopic approach compared to the open one during the first postoperative month [MD = −4.47; 95%CI: 7.24: 1.70]; however, this difference was deemed insignificant over the short- and long-terms (Fig. S1).

Excellent recovery, defined as DASH score <20, was similar between both arthroscopic and open methods [4 studies, logOR = 0.33; 95%CI: 0.09: 0.75] (Fig. S2).

#### 3.4.2 MEPS score

The meta-analysis revealed no significant difference in postoperative MEPS score between the arthroscopic and open approaches [3 studies, MD = 0.31; 95%CI: 2.33: 2.95] (Fig. S3). This finding was supported by the lack of heterogeneity [I^2^ = 0 %; *P* = 0.87].

#### 3.4.3 Good/excellent outcome

Six studies examined the rate of good-to-excellent recovery after surgery. The meta-analysis; however, showed no significant difference in good-to-excellent recovery between the arthroscopic and open methods [logOR = 0.36; 95%CI: 0.09: 0.81] (Fig. S4). This finding was supported by the lack of heterogeneity [I^2^ = 0 %; *P* = 0.68].

#### 3.4.4 Poor outcome/unfavorable results

Four studies examined the rate of poor or unfavorable recovery after surgery. The meta-analysis; however, showed no significant difference in poor recovery between the arthroscopic and open methods [logOR = 0.43; 95%CI: 0.42: 1.27] (Fig. S5). This finding was supported by the lack of heterogeneity [I^2^ = 0 %; *P* = 0.52].

#### 3.4.5 Surgical failure

Three studies examined the rate of surgical failure after the management of lateral epicondylitis. The meta-analysis; however, showed no significant difference in the odds of failure between the arthroscopic and open methods [logOR = 0.31; 95%CI: 0.72: 1.34] (Fig. S6). This finding was supported by the lack of heterogeneity [I^2^ = 0 %; *P* = 0.56].

#### 3.4.6 Revision or additional surgical treatment

Seven studies examined the need for revision or additional surgery for the management of lateral epicondylitis. The meta-analysis; however, showed no significant difference in the odds of revision surgery between the arthroscopic and open methods [logOR = 0.31; 95%CI: 0.08: 0.69] (Fig. S7). This finding was supported by the lack of heterogeneity [I^2^ = 0 %; *P* = 0.74].

### 3.5 Elbow parameters

#### 3.5.1 ROM (flexion – extension)

Three studies examined the range of motion of the elbow (particularly during flexion/extension) following the management of lateral epicondylitis. The meta-analysis; however, showed no significant difference in the elbow ROM between the arthroscopic and open methods [logOR = 0.47; 95%CI: 0.30: 1.24] (Fig. S8). This finding was supported by the lack of heterogeneity [I^2^ = 0 %; *P* = 0.37].

#### 3.5.2 Grip strength (kg)

Six studies examined the grip strength following the management of lateral epicondylitis. The meta-analysis; however, showed no significant difference in grip strength between the arthroscopic and open methods [logOR = 0.49; 95%CI: 0.43: 1.41] (Fig. 5). This finding was supported by the insignificantly low heterogeneity level [I^2^ = 23.99 %; *P* = 0.25].

**Fig. 5:**
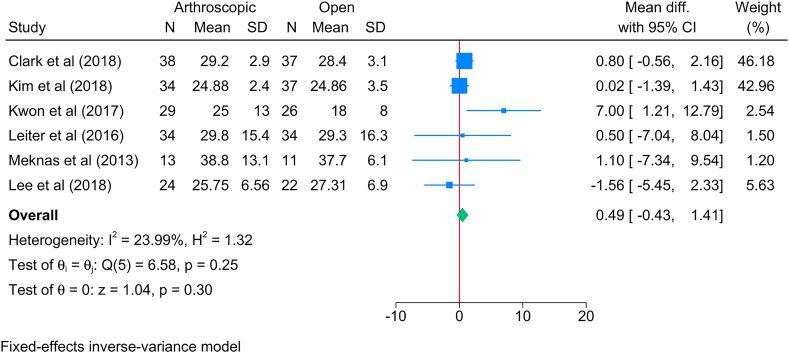
Forest plot showing the difference in grip strength between arthroscopic and open lateral release in patients with lateral epicondylitis.

The subgroup analysis based on time showed only a significant reduction in grip strength in favor of the arthroscopic group compared to the open group at 6 months of follow-up [MD = −1.50; 95%CI: 2.67: 0.33] (Fig. S9). However, no significant change was observed beyond that timepoint until 60 months of follow-up.

### 3.6 Associated radiographic findings

#### 3.6.1 Intra-articular loose bodies

Two studies examined the presence of intra-articular loose bodies following the surgical management of lateral epicondylitis. The meta-analysis; however, showed no significant difference in the odds of intra-articular loose bodies between the arthroscopic and open methods [logOR = 1.47; 95%CI: 0.38: 3.31] (Fig. S10). This finding was supported by the insignificantly low heterogeneity level [I^2^ = 23.99 %; *P* = 0.98].

#### 3.6.2 Postoperative synovitis

Two studies examined the presence of synovitis following the surgical management of lateral epicondylitis. The meta-analysis; however, showed no significant difference in the odds of synovitis between the arthroscopic and open methods [logOR = 1.72; 95%CI: 1.89: 5.33] (Fig. S11). Although significant heterogeneity was found [I^2^ = 77.10 %; *P* = 0.04], a sensitivity analysis was not feasible due to the small sample size.

### 3.7 Postoperative pain

#### 3.7.1 VAS measures

The overall VAS score was reported by 10 studies. The meta-analysis; however, showed no significant difference in the total VAS score between the arthroscopic and open methods [MD = −0.14; 95 % CI: 0.34: 0.06]. This finding was supported by the lack of statistical heterogeneity [I^2^ = 0 %; *P* = 0.85] (Fig. 6). This was consistent with VAS measurement during rest [2 studies, MD = 0.18; 95%CI: 0.11: 0.47] (Fig. S12) and during work [2 studies, MD = 0.14; 95%CI: 0.12: 0.41] (Fig. S13), where no significant difference was observed between both methods.

**Fig. 6:**
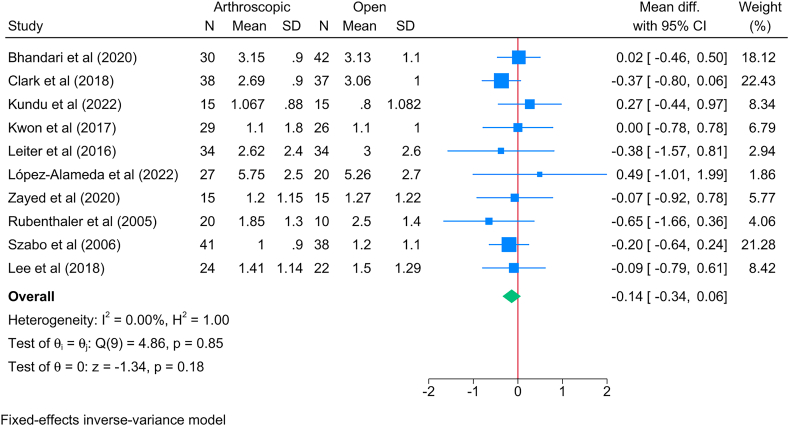
Forest plot showing the difference in pain score (measured by visual analogue scale) between arthroscopic and open lateral release in patients with lateral epicondylitis.

The subgroup analysis of the main VAS analysis showed a significant reduction in VAS score in favor of the arthroscopic method only by the 1st month [2 studies, MD = −1.13; 95 % CI: 1.77: 0.50] (Fig. S14). However, beyond this timepoint, both methods had similar scores.

#### 3.7.2 Recurrence of pain

Two studies examined the recurrence of pain following the surgical management of lateral epicondylitis. The meta-analysis; however, showed no significant difference in the odds of pain recurrence between the arthroscopic and open methods [logOR = 0.01; 95%CI: 2.16: 2.17] (Fig. S15). Although a moderate level of heterogeneity was observed, it was statistically insignificant [I^2^ = 67.79 %; *P* = 0.08].

### 3.8 Work-related outcomes

#### 3.8.1 Return to work (months)

Four studies focused on the duration before patients could resume work after undergoing surgery for lateral epicondylitis. The meta-analysis indicated a notable decrease in the time required to return to work when using the arthroscopic technique [MD = −1.64 months; 95%CI: 2.60 to −0.68] (Fig. S16). There was a moderate degree of heterogeneity observed [I^2^ = 63.19 %; *P* = 0.04]; nonetheless, sensitivity analyses showed that the estimate remained consistent even after individually removing each study from the analysis.

#### 3.8.2 Time taken off work (weeks)

Two studies examined the time taken off work following the surgical management of lateral epicondylitis. The meta-analysis showed no significant change in the time taken off work between both the arthroscopic and open methods [MD = −0.07; 95 % CI: 0.61: 0.47] (Fig. S17). This finding was supported by the lack of heterogeneity [I^2^ = 0 %; *P* = 0.42].

### 3.9 Other clinical outcomes

#### 3.9.1 Operative time (mins)

Seven studies examined the operative time taken for the surgical management of lateral epicondylitis. The meta-analysis showed a significant increase in the operative time in favor of the arthroscopic approach compared to the open one [MD = 8.94 min; 95 % CI: 0.08: 17.79] (Fig. S18). A considerable amount of significant heterogeneity was noted [I^2^ = 99.10 %; *P* = 0.001]. The sensitivity analysis revealed a significant change in the reported estimate with excluding each study separately (Fig. S19).

#### 3.9.2 Postoperative complications

Nine studies examined the rate of postoperative complications following the surgical management of lateral epicondylitis. The meta-analysis showed no significant change in the odds of complications between both the arthroscopic and open methods [logOR = −0.28; 95 % CI: 0.70: 0.14] (Fig. S20). This finding was supported by the lack of heterogeneity [I^2^ = 0 %; *P* = 0.98].

## 4 Discussion

### 4.1 Comparison of functional recovery outcomes

Our meta-analysis provides a comprehensive comparison of arthroscopic versus open release for lateral epicondylitis, particularly focusing on functional recovery as measured by the DASH and MEPS scores. Consistent with prior reviews,^6^^,^^8^ we found no significant difference in overall postoperative DASH and MEPS scores between the two methods. These findings suggest that both surgical approaches are effective in restoring function without any marked superiority of one over the other.^6^ Determinants of functional recovery are diverse, including a wide variety of factors. For instance, patients with severe preoperative limitations typically show more significant improvements post-treatment but may still have suboptimal DASH scores compared to those with milder baseline symptoms. Additionally, compliance with structured, patient-specific rehabilitation protocols leads to better functional recovery and higher DASH scores. Furthermore, recovery can vary due to age, gender, and occupation, with older adults typically experiencing slower recovery and physically demanding jobs requiring additional rehabilitation for work-specific activities. Coexisting musculoskeletal or systemic conditions can complicate postoperative rehabilitation, potentially resulting in reduced DASH scores. Finally, the surgeon's proficiency with the chosen technique and the occurrence of complications like infections or nerve injuries can prolong recovery and impact DASH scores. Unfortunately, the impact of these factors on functional recovery could not be investigated due to the nature of reported data. Therefore, future studies should address these points.

Interestingly, the subgroup analysis revealed a significant reduction in DASH scores favoring the arthroscopic approach during the first postoperative month, indicating a potentially quicker initial recovery. This aspect might be particularly appealing to patients and surgeons aiming for a faster return to daily activities, a finding that aligns with some studies highlighted in the review articles which emphasized the importance of early functional recovery in determining surgical success.

### 4.2 Surgical outcomes and complications

Both procedures risk damaging nearby nerves, particularly the radial nerve. This can result in transient or persistent sensory deficits and, occasionally, motor weakness. Arthroscopic techniques tend to carry a lower risk due to smaller incisions, but the risk remains due to the proximity of nerves in the operative field. Our analysis found no significant differences in the rates of surgical failures and revisions between the two methods, highlighting the comparable long-term dependability of both techniques. Synovitis and intra-articular loose bodies can develop or persist post-surgery, potentially requiring further arthroscopic debridement, and this was evident in our analysis. The comparable rates of postoperative complications, as we observed, also support the assertion made in earlier studies that the choice of technique should be tailored to the surgeon's expertise and patient-specific factors rather than inherent risks associated with either method.^6^^,^^15^

### 4.3 Elbow motion and grip strength

Grip strength is another key metric in assessing functional recovery, reflecting the forearm muscle's ability to generate force. In the short term, arthroscopic surgery may confer a temporary advantage in grip strength at six months postoperatively. This could be due to the less invasive nature of the procedure, which minimizes soft tissue disruption and allows for quicker initial recovery. The equivalence in elbow range of motion (ROM) and grip strength outcomes further supports the parity between arthroscopic and open releases. Although arthroscopic release showed a temporary advantage in grip strength at the 6-month follow-up, this did not persist into longer-term follow-ups, suggesting that any early benefits may diminish over time. This transient benefit might be related to less invasive nature of the arthroscopic approach, which potentially allows for lesser tissue disruption.^6^^,^^41^

### 4.4 Radiographic and pain assessment outcomes

Our findings regarding intra-articular loose bodies and evident synovitis did not show significant differences, pointing to a negligible impact of surgical choice on these specific radiographic outcomes.

During the immediate postoperative period, patients who undergo arthroscopic lateral release tend to report reduced immediate postoperative pain compared to open surgery. This may be due to the minimally invasive nature of the procedure, leading to less disruption of surrounding tissues. Smaller incisions and less soft tissue dissection reduce the trauma and inflammation that contribute to early postoperative pain.^15^ On the other hand, open surgery requires a larger incision and more extensive dissection of soft tissue to access the affected area. This often results in greater immediate postoperative pain due to increased tissue damage and inflammation. Proper pain management and early mobilization can help alleviate this pain, but it generally remains higher than with arthroscopic techniques.

In the medium- and long-term, arthroscopic surgery can lead to faster pain resolution, as demonstrated by significantly lower VAS pain scores within the first postoperative month. However, beyond this period, pain differences between arthroscopic and open approaches diminish. Both techniques achieve comparable long-term pain control, indicating that the initial arthroscopic benefits may not persist. On the other hand, open surgery also provides effective long-term pain relief, although it may take longer for pain to subside compared to arthroscopic surgery. Over time, with consistent rehabilitation, patients achieve similar pain reduction, highlighting the importance of proper postoperative care.^15^

In terms of recurrent of pain, both methods are associated with a low recurrence rate of pain following lateral release. The risk of recurrence is linked to surgical failures or incomplete resolution of the tendinosis, which is uncommon with either method. Proper patient selection and adherence to postoperative rehabilitation protocols are crucial to minimizing this risk.^15^

### 4.5 Work-related outcomes

The significant reduction in the time taken to return to work with the arthroscopic approach is a critical finding, considering the economic implications and patient satisfaction. The significant reduction in the time taken to return to work following the arthroscopic approach is a noteworthy finding with broader implications, especially in terms of economic considerations and patient satisfaction. A shorter recovery period allows patients to resume their professional activities sooner, which is crucial for maintaining economic stability and minimizing the financial impact of extended medical leave. This is particularly important for individuals employed in physically demanding professions where time away from work can lead to substantial income loss.

Faster return to work not only benefits the patients but also their employers, who face reduced costs associated with temporary staffing and productivity losses. Moreover, a quicker recovery and resumption of professional activities contribute to improved morale and consistent work quality within the workplace. From the healthcare perspective, shorter recovery times reduce the economic burden on healthcare systems through shorter hospital stays and decreased need for extended rehabilitation, leading to overall cost savings.

The advantages observed with the arthroscopic approach support the hypothesis that less invasive procedures facilitate quicker rehabilitation. Although arthroscopic surgery may involve marginally longer operative times, its benefits in reducing postoperative recovery time are significant. This approach leads to early functional recovery, allowing patients to regain full productivity swiftly, which is a crucial factor in the overall evaluation of surgical success.^6^

### 4.6 Strengths and limitations

Our systematic review and meta-analysis is strengthened by several factors. Firstly, the comprehensive literature search and rigorous selection criteria ensured a thorough exploration of available studies, enhancing the generalizability of our findings. Additionally, the inclusion of multiple study designs, such as RCTs, prospective cohorts, and retrospective cohorts, provides a broad perspective on the outcomes of interest. Furthermore, we included non-English studies to ensure adequate reporting while minimizing the risk of selection and publication bias considerably.

However, our study is not without limitations. The heterogeneity observed in some outcomes, particularly in subgroup analyses, suggests variability in study design, participant characteristics, or surgical techniques that could affect the results. The presence of moderate-to-high risk of bias in some included studies also tempers the strength of our conclusions. Furthermore, the limited number of studies reporting long-term follow-up data restricts our understanding of the durability of surgical outcomes over time.

### 4.7 Literature gaps and future research directions

Our review has identified several critical outcomes, yet there are notable deficiencies in the existing research. Specifically, there is a scarcity of information on patient-reported outcomes like quality of life and satisfaction after surgery. Moreover, the majority of studies we analyzed originated from higher-income nations, which may restrict the relevance of our findings to contexts with fewer resources. Additionally, we did not restrict the search to publications in the past two decades, as recommended in recent guidelines.^17^ This could account, to some extent, to the observed heterogeneity given the progressive advancement of the surgical management of lateral epicondylitis. Moreover, only a few studies investigated revision rate between both procedures; this needs to be addressed thoroughly in future research to determine long-term efficacy.

Future research should aim to address these gaps by including more diverse populations and incorporating patient-centered outcomes. Long-term follow-up studies are also needed to better understand the durability of both surgical techniques. Moreover, further investigation into the cost-effectiveness of arthroscopic versus open release could inform healthcare policy and surgical practice, ensuring that resource allocation aligns with patient benefits.

## 5 Conclusion

Our systematic review and meta-analysis confirm that both arthroscopic and open release techniques are effective surgical treatments for lateral epicondylitis, offering similar long-term results. However, the choice of procedure might affect the initial postoperative recovery, suggesting arthroscopy might be better for patients who need a faster functional recovery and a quicker return to work. Further research, ideally through multicenter randomized controlled trials, could clarify these results, aiming to reduce bias and investigate the reasons behind the differences in short-term outcomes.

## Author contribution

**Maher Ghandour:** Conceptualization, formal analysis, investigation, methodology, and Writing – review & editing. **Bashour Hanna and Tanios Dagher**: Supervision, project administration, software, resources, validation, visualization, and Writing – review & editing. **Diaa Al Salloum,** Mohamad Houssein JABER, Ghadi Abou Orm, Ali Ghosn, Sadek Jaber, Hicham Abd El **Nour, and Anthony Chalfoun:** Investigation, methodology, data curation, visualization, and Writing – original draft.

## Funding

No funding was provided for this research.

## Guardian/patient's consent

This research did not include any human subjects. Therefore, the need for patient consent was not required.

## Ethical statement

Given the nature of this research, the need for ethical approval was waived.

## Data availability statement

The datasets generated during and/or analyzed during the current study are available from the corresponding author upon reasonable request.

## Declaration of competing interest

All of the study authors declare no competing interested associated with the conduct of this research.

## Figures and Tables

**figs1:**
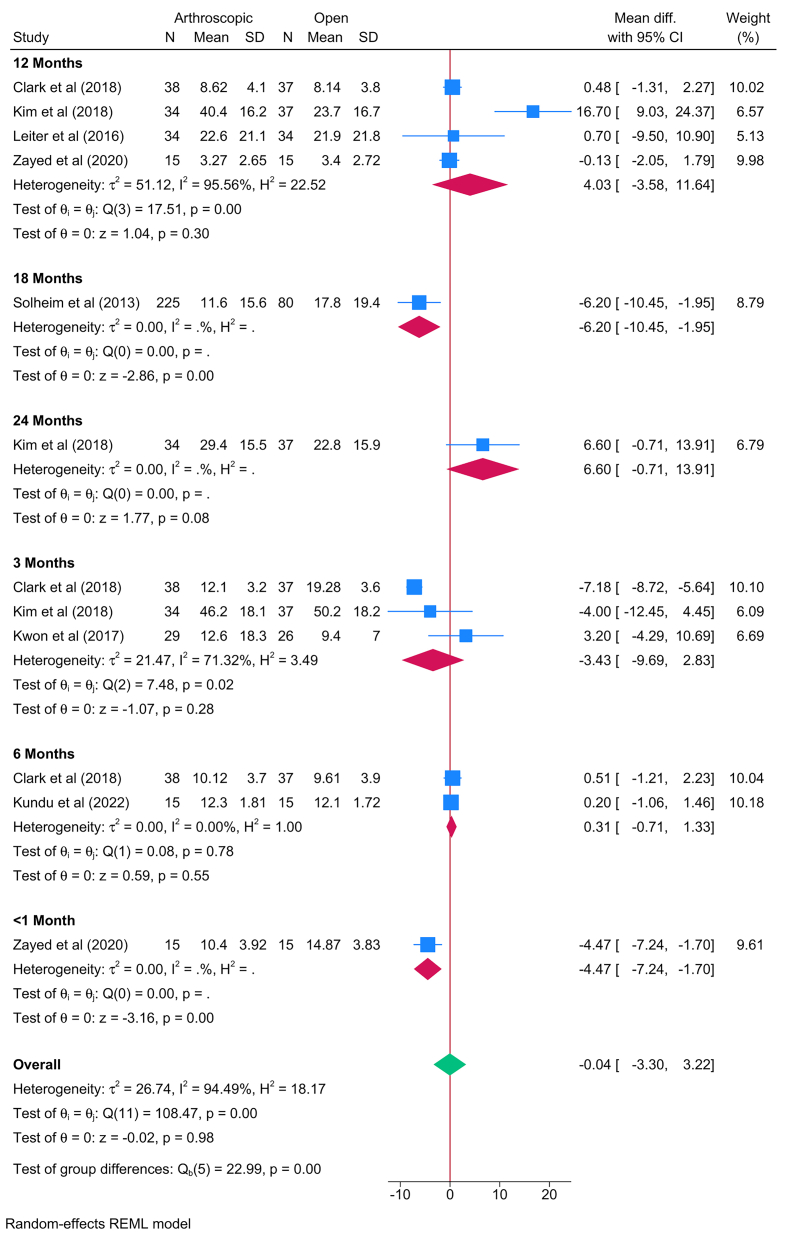


**figs2:**
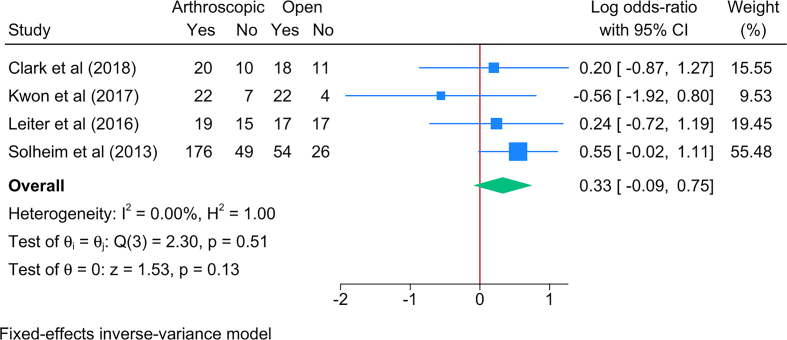


**figs3:**
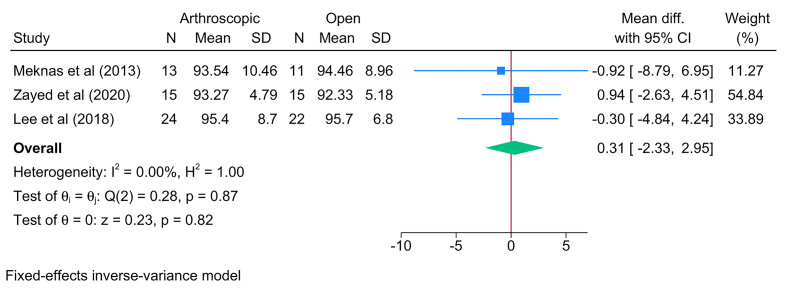


**figs4:**
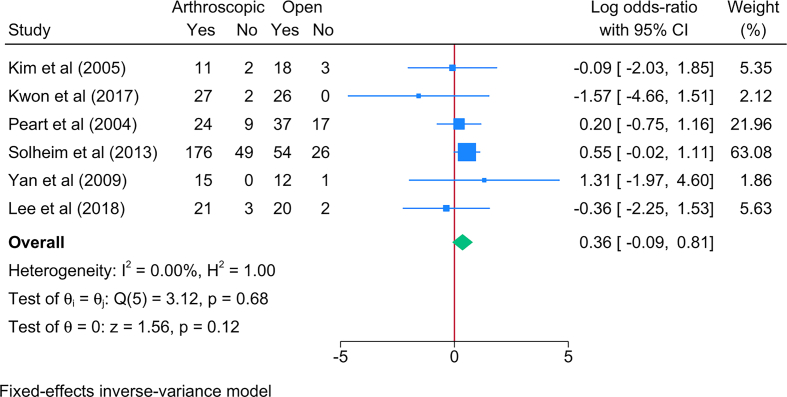


**figs5:**
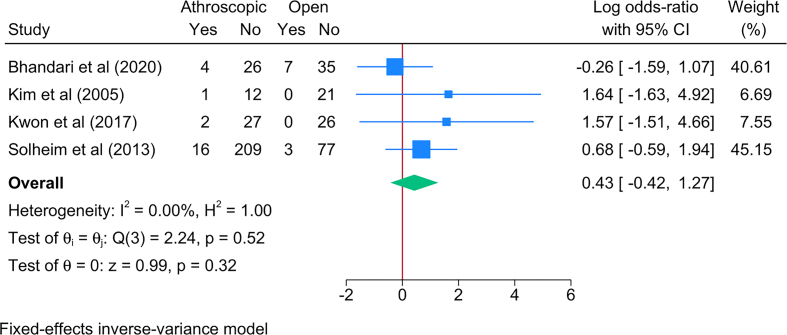


**figs6:**
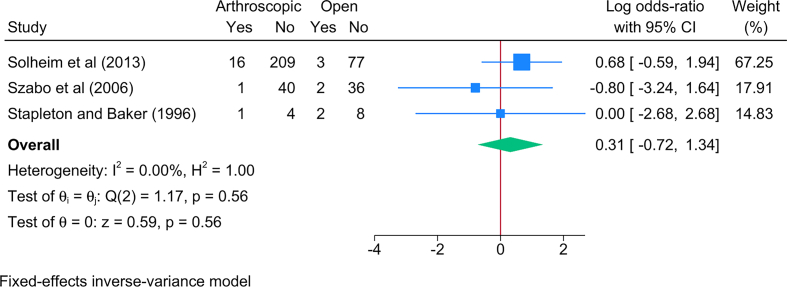


**figs7:**
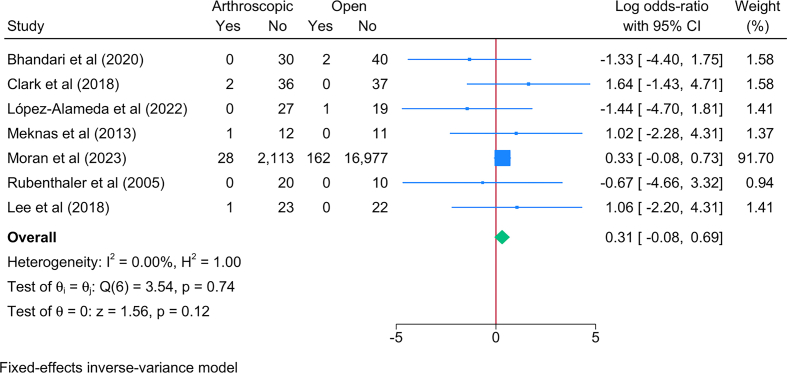


**figs8:**
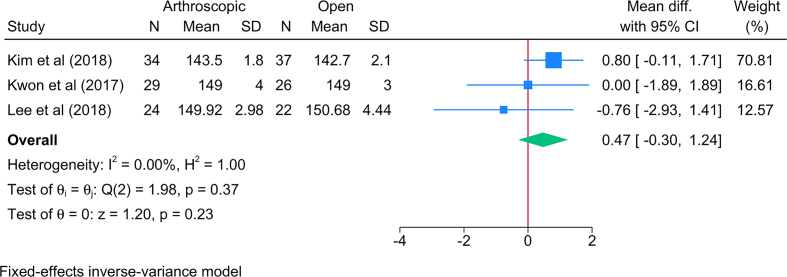


**figs9:**
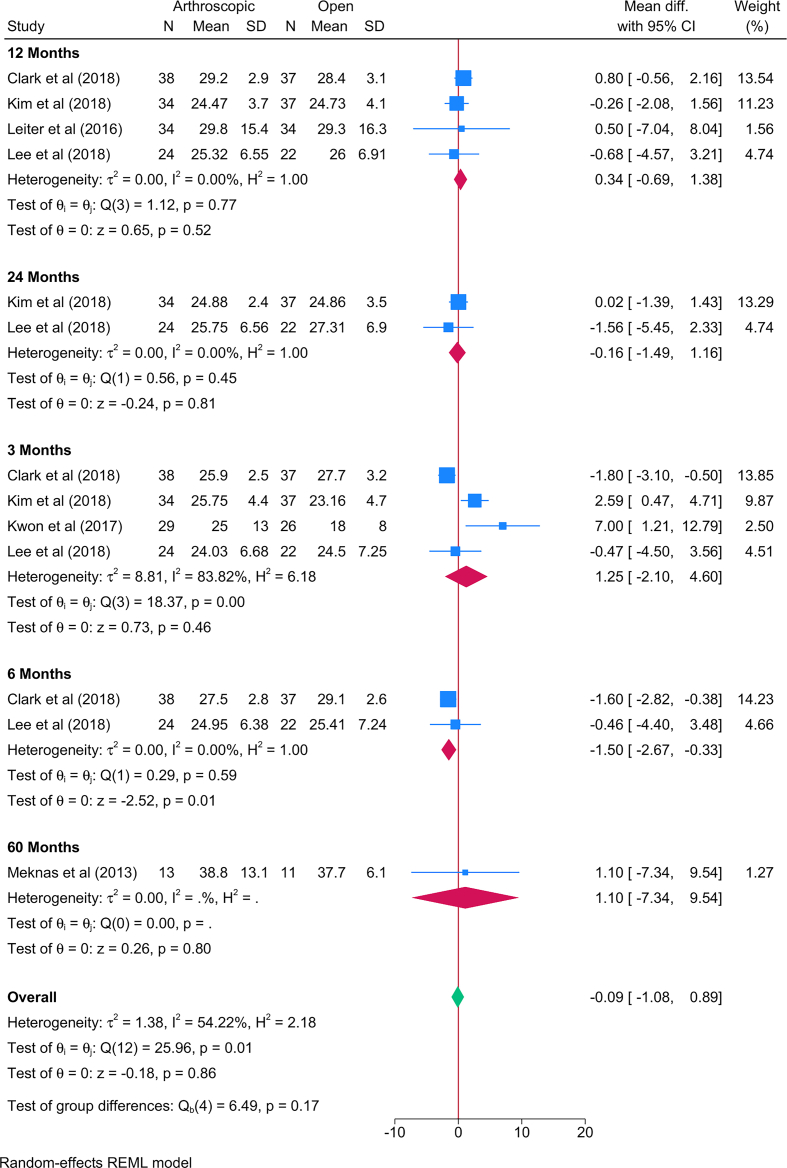


**figs10:**
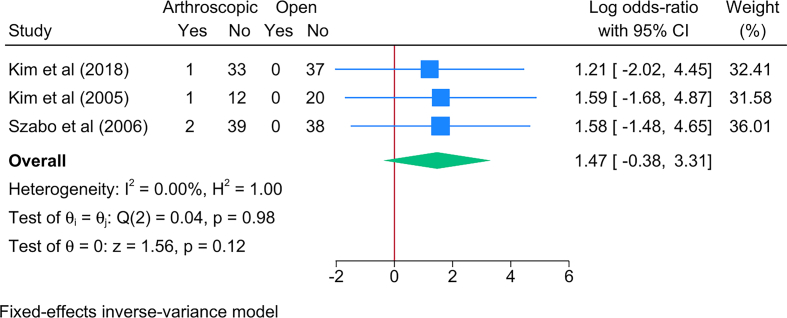


**figs11:**
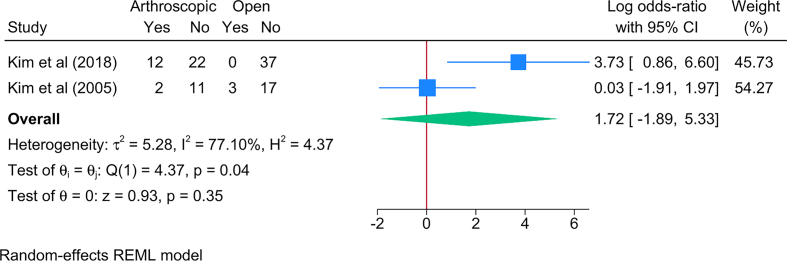


**figs12:**
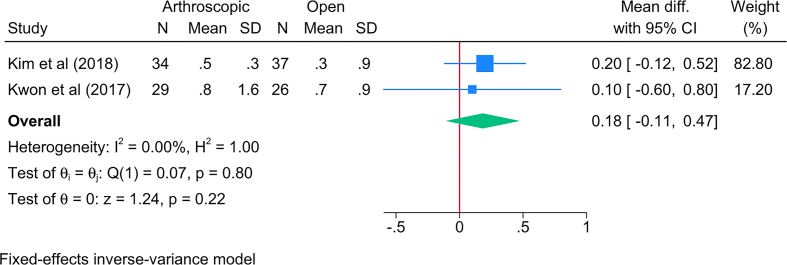


**figs13:**
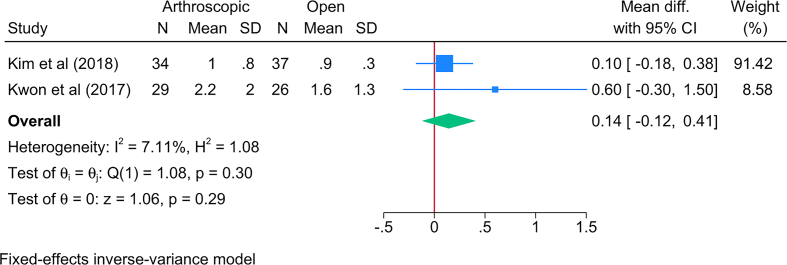


**figs14:**
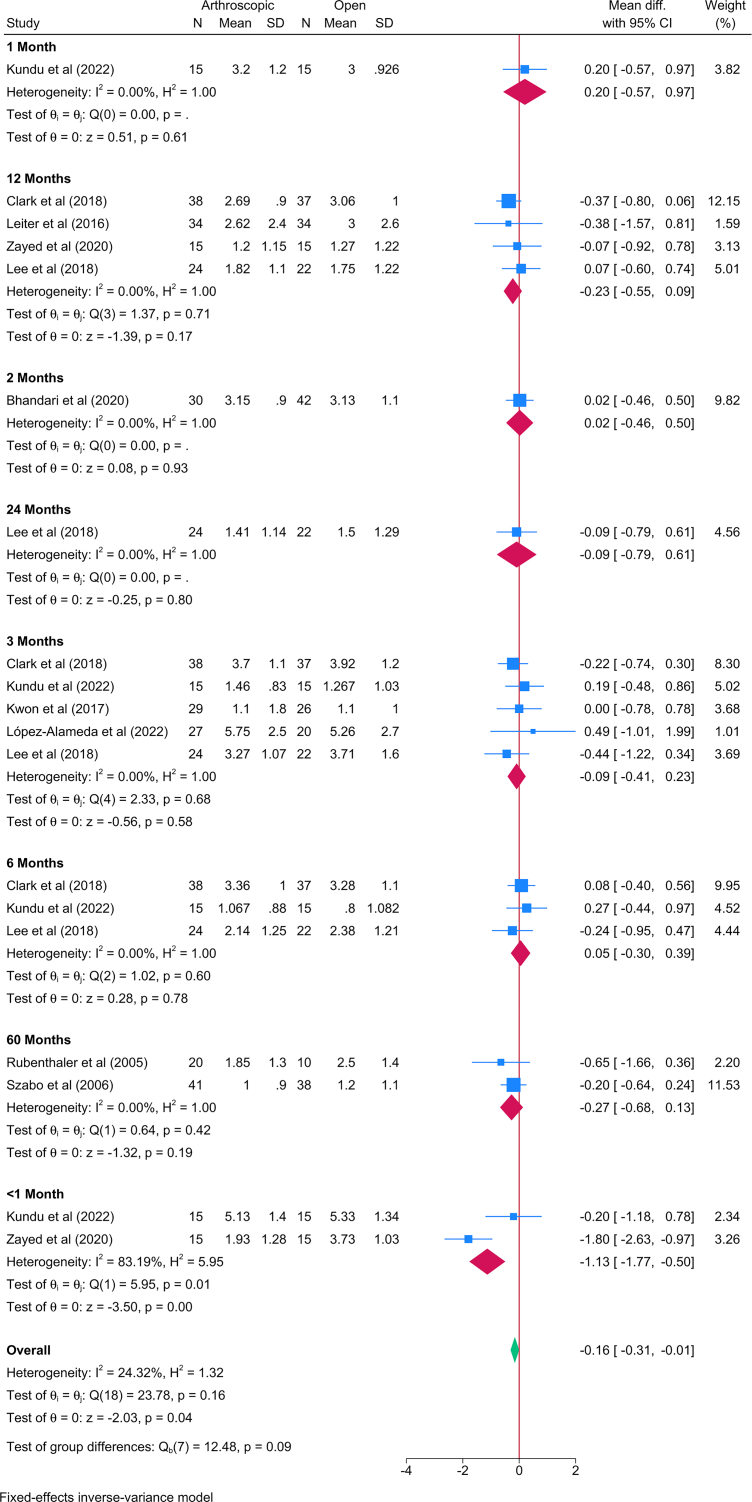


**figs15:**
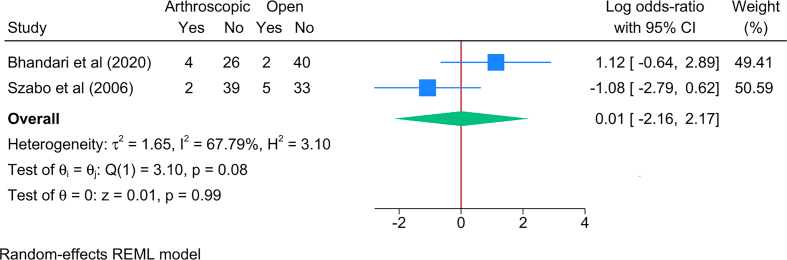


**figs16:**
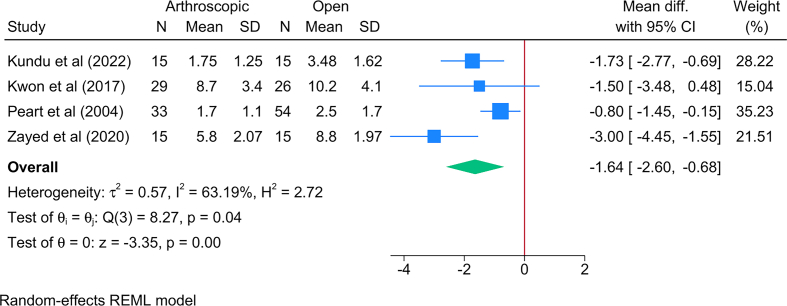


**figs17:**
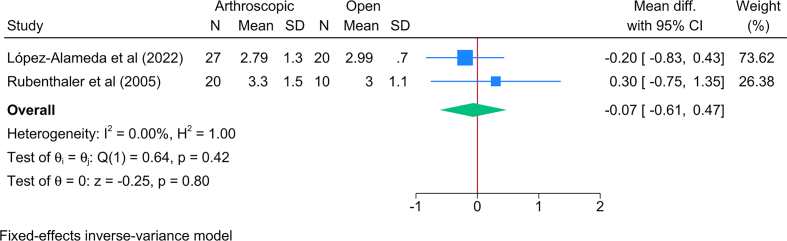


**figs18:**
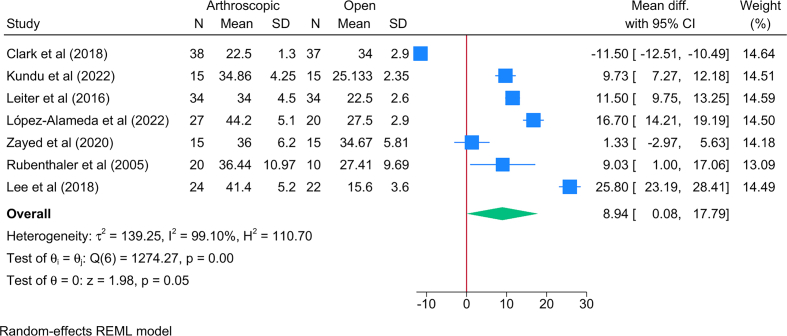


**figs19:**
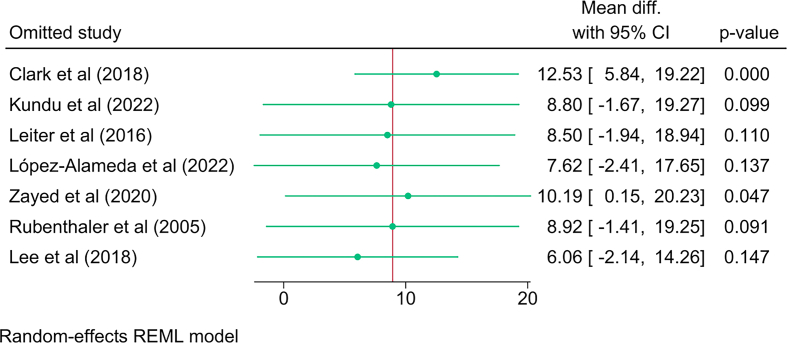


**figs20:**
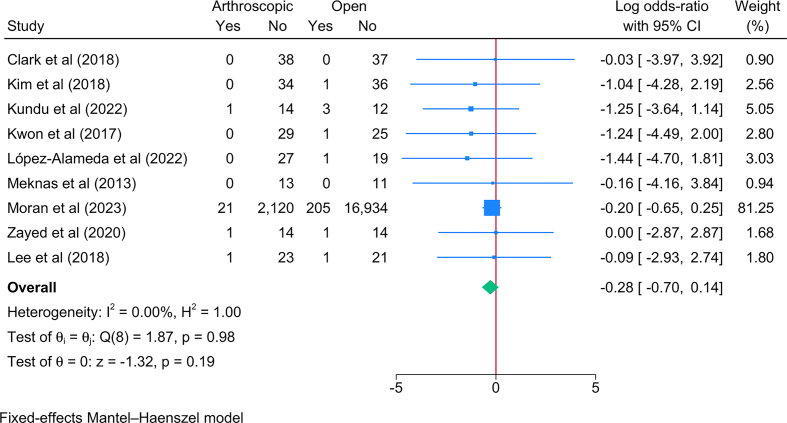


## References

[1] Ikonen J., Lähdeoja T., Ardern C.L., Buchbinder R., Reito A., Karjalainen T. (2022). Persistent tennis elbow symptoms have little prognostic value: a systematic review and meta-analysis. Clin Orthop Relat Res.

[2] Descatha A., Albo F., Leclerc A. (2016). Lateral epicondylitis and physical exposure at work? A review of prospective studies and meta‐analysis. Arthritis Care Res.

[3] Walz D.M., Newman J.S., Konin G.P., Ross G. (2010). Epicondylitis: pathogenesis, imaging, and treatment. Radiographics.

[4] Potter H.G., Hannafin J.A., Morwessel R.M., DiCarlo E.F., O'Brien S.J., Altchek D.W. (1995). Lateral epicondylitis: correlation of MR imaging, surgical, and histopathologic findings. Radiology.

[5] Karabinov V., Georgiev G.P. (2022). Lateral epicondylitis: new trends and challenges in treatment. World J Orthoped.

[6] Li Y., Guo S., Li S., Yang G., Lu Y. (2023). Is there any difference in clinical outcome between open and arthroscopic treatment for tennis elbow? A systematic review and meta‐analysis. Orthop Surg.

[7] Lo M.Y., Safran M.R. (2007). Surgical treatment of lateral epicondylitis: a systematic review. Clin Orthop Relat Res.

[8] Wang W., Chen J., Lou J., Shentu G., Xu G. (2019). Comparison of arthroscopic debridement and open debridement in the management of lateral epicondylitis: a systematic review and meta-analysis. Medicine.

[9] Santiago A.O., Rios-Russo J.L., Baerga L., Micheo W. (2021). Evidenced-based management of tennis elbow. Current Physical Medicine and Rehabilitation Reports.

[10] Babaqi A.A., Kotb M.M., Said H.G., AbdelHamid M.M., ElKady H.A., ElAssal M.A. (2014). Short-term evaluation of arthroscopic management of tennis elbow; including resection of radio-capitellar capsular complex. J Orthop.

[11] Solheim E., Hegna J., Øyen J. (2013). Arthroscopic versus open tennis elbow release: 3-to 6-year results of a case-control series of 305 elbows. Arthrosc J Arthrosc Relat Surg.

[12] Yan H., Cui G.-Q., Liu Y.-L., Xiao J., Yang Y.-P., Ao Y.-F. (2009). A randomized comparison of open and arthroscopic Nirschl debridement for refractory lateral epicondylitis. Zhonghua wai ke za zhi [Chinese Journal of Surgery].

[13] Kundu B., Kumar D. (2022). Comparative study of functional outcomes of open versus arthroscopic surgery of lateral epicondylitis in a tertiary care hospital. International Surgery Journal.

[14] Kwon B.C., Kim J.Y., Park K.-T. (2017). The Nirschl procedure versus arthroscopic extensor carpi radialis brevis débridement for lateral epicondylitis. J Shoulder Elbow Surg.

[15] Pierce T.P., Issa K., Gilbert B.T. (2017). A systematic review of tennis elbow surgery: open versus arthroscopic versus percutaneous release of the common extensor origin. Arthrosc J Arthrosc Relat Surg.

[16] Page M.J., McKenzie J.E., Bossuyt P.M. (2021). The PRISMA 2020 statement: an updated guideline for reporting systematic reviews. Br Med J.

[17] Muka T., Glisic M., Milic J. (2020). A 24-step guide on how to design, conduct, and successfully publish a systematic review and meta-analysis in medical research. Eur J Epidemiol.

[18] Abdelaal A., Eltaras M.M., Katamesh B.E. (2023). The prevalence and presentation patterns of microcystic macular oedema: a systematic review and meta-analysis of 2128 glaucomatous eyes. Eye.

[19] Amir-Behghadami M., Janati A. (2020). Population, Intervention, Comparison, Outcomes and Study (PICOS) design as a framework to formulate eligibility criteria in systematic reviews. Emerg Med J.

[20] Koca K., Erdem Y., Neyişci Ç., Erşen Ö. (2017). Intramedullary elastic nailing of the displaced radial neck fractures in children. Acta Orthop Traumatol Turcica.

[21] Phadnis J., Trompeter A., Gallagher K., Bradshaw L., Elliott D.S., Newman K.J. (2012). Mid-term functional outcome after the internal fixation of distal radius fractures. J Orthop Surg Res.

[22] Luo D., Wan X., Liu J., Tong T. (2018). Optimally estimating the sample mean from the sample size, median, mid-range, and/or mid-quartile range. Stat Methods Med Res.

[23] Wan X., Wang W., Liu J., Tong T. (2014). Estimating the sample mean and standard deviation from the sample size, median, range and/or interquartile range. BMC Med Res Methodol.

[24] Mavridis D., Salanti G., Furukawa T.A., Cipriani A., Chaimani A., White I.R. (2019). Allowing for uncertainty due to missing and LOCF imputed outcomes in meta‐analysis. Stat Med.

[25] Sedgwick P. (2013). Meta-analyses: heterogeneity and subgroup analysis. Br Med J.

[26] Bhandari L., Bouri F., Ozyurekoglu T. (2020). Open versus arthroscopic treatment of chronic lateral epicondylitis and worker's compensation. Arthroscopy, Sports Medicine, and Rehabilitation.

[27] Clark T., McRae S., Leiter J., Zhang Y., Dubberley J., MacDonald P. (2018). Arthroscopic versus open lateral release for the treatment of lateral epicondylitis: a prospective randomized controlled trial. Arthrosc J Arthrosc Relat Surg.

[28] Kim D.S., Chung H.J., Yi C.-H., Kim S.-H. (2018). Comparison of the clinical outcomes of open surgery versus arthroscopic surgery for chronic refractory lateral epicondylitis of the elbow. Orthopedics.

[29] Kim Y.-G., Lee J.-H., Gwak J.-H. (2005). The Academic Congress of Korean Shoulder and Elbow Society.

[30] Kim Y.-K., Lee J.-H., Kwak J.-H., Moon S.-H. (2008). Recalcitrant lateral epicondylitis: open and arthroscopic release. Journal of the Korean Orthopaedic Association.

[31] Lee J.-H., Park I., Hyun H.-S., Shin S.-J. (2018). A comparison of radiofrequency-based microtenotomy and arthroscopic release of the extensor carpi radialis brevis tendon in recalcitrant lateral epicondylitis: a prospective randomized controlled study. Arthrosc J Arthrosc Relat Surg.

[32] Leiter J., Clark T., McRae S., Dubberley J., MacDonald P.B. (2016). Open versus arthroscopic tennis elbow release: randomized controlled trial. Orthopaedic Journal of Sports Medicine.

[33] López-Alameda S., Varillas-Delgado D., De Felipe-Gallego J., González-Granados M.G., Hernández-Castillejo L.E., García-de Lucas F. (2022). Arthroscopic surgery versus open surgery for lateral epicondylitis in an active work population: a comparative study. J Shoulder Elbow Surg.

[34] Meknas K., Al Hassoni T.N., Odden-Miland Å., Castillejo M., Kartus J. (2013). Medium-term results after treatment of recalcitrant lateral epicondylitis: a prospective, randomized study comparing open release and radiofrequency microtenotomy. Orthopaedic Journal of Sports Medicine.

[35] Moran J., Gillinov S.M., Jimenez A.E. (2023). No difference in complication or reoperation rates between arthroscopic and open debridement for lateral epicondylitis: a national database study. Arthrosc J Arthrosc Relat Surg.

[36] Peart R.E., Strickler S.S., Schweitzer Jr KM. (2004). Lateral epicondylitis: a comparative study of open and arthroscopic lateral release. Am J Orthoped.

[37] Rubenthaler F., Wiese M., Senge A., Keller L., Wittenberg R.H. (2005). Long-term follow-up of open and endoscopic Hohmann procedures for lateral epicondylitis. Arthrosc J Arthrosc Relat Surg.

[38] Stapleton T., Baker C. (1996). Arthroscopic treatment of lateral epicondylitis: a clinical study. Arthroscopy.

[39] Szabo S.J., Savoie I.I.I.F.H., Field L.D., Ramsey J.R., Hosemann C.D. (2006). Tendinosis of the extensor carpi radialis brevis: an evaluation of three methods of operative treatment. J Shoulder Elbow Surg.

[40] Zayed F.H. (2020). Comparative study: arthroscopic versus open tennis elbow release. Al-Azhar International Medical Journal..

[41] Burn M.B., Mitchell R.J., Liberman S.R., Lintner D.M., Harris J.D., McCulloch P.C. (2018). Open, arthroscopic, and percutaneous surgical treatment of lateral epicondylitis: a systematic review. Hand.

