# Supplementary material for: A comparative meta-analysis of the efficacy and safety of arthroscopic versus open surgery in patients with lateral epicondylitis
Source: J Orthop. 2024 Jul 25;59:41–50. doi: 10.1016/j.jor.2024.07.018 (PMC11439539; doi:10.1016/j.jor.2024.07.018)
Supplement: Multimedia component 1 [file mmc1.docx]

**Table S1.** The detailed search strategy used in the database search of this systematic review

| Database | No | Search Query | Results |
| --- | --- | --- | --- |
| PubMed [Date of search: 22/2/2024] | | | |
|  | #1 | “lateral epicondylitis”[tiab] OR “tennis elbow”[tiab] OR “lateral humeral epicondylitis”[tiab] OR "Tennis Elbow"[Mesh] | 2715 |
|  | #2 | Open[tiab] | 643421 |
|  | #3 | Arthroscopic*[tiab] | 30607 |
|  | #4 | #1 AND #2 AND #3 | 62 |
| Scopus [Date of search: 22/2/2024] | | | |
|  | #1 | TITLE-ABS-KEY (“lateral epicondylitis”) OR TITLE-ABS-KEY (“tennis elbow”) OR TITLE-ABS-KEY (“lateral humeral epicondylitis”) | 4202 |
|  | #2 | TITLE-ABS-KEY (Open) | 1911087 |
|  | #3 | TITLE-ABS-KEY (Arthroscopic*) | 40025 |
|  | #4 | #1 AND #2 AND #3 | 101 |
| Web of Science [Date of search: 22/2/2024] | | | |
|  | #1 | AB=“lateral epicondylitis” OR AB=“tennis elbow” OR AB=“lateral humeral epicondylitis” | 1469 |
|  | #2 | AB=Open | 1758929 |
|  | #3 | AB=Arthroscopic* | 24136 |
|  | #4 | #1 AND #2 AND #3 | 48 |
| Cochrane Library of Randomized Trials [Date of search: 22/2/2024] | | | |
|  | #1 | “lateral epicondylitis” OR “tennis elbow” OR “lateral humeral epicondylitis” | 1188 |
|  | #2 | Open | 177184 |
|  | #3 | Arthroscopic* | 4640 |
|  | #4 | #1 AND #2 AND #3 [Trials only] | 13 |
| Google Scholar [Date of search: 22/2/2024] | | | |
|  | With all of the words | Open arthroscopic lateral epicondylitis | - |
|  | With the exact phrase | - | - |
|  | With at least one of the words | - | - |
|  | Total | As per guidelines, only the first 200 records were screened | 200 |
